# Bioglycans and Natural Glycosides As a Promising Research Topic in Bioorganic Chemistriy

**Published:** 2010-07

**Authors:** Yu.S. Ovodov

**Affiliations:** Institute of Physiology, Komi Science Center, The Urals Branch, Russian Academy of Sciences

**Keywords:** bioglycans, natural glycosides, low-molecular-weight bioregulators

## Abstract

This review defines bioorganic chemistry as one of the most important constituents of physico–chemical biology, which is a fundamental life science. The problems and goals of bioorganic chemistry are examined through a comparatively small number of examples. Bioorganic chemistry is supposed to be a logical continuation of the chemistry of the natural substances that arose many years ago. Bioorganic chemistry has contributed some achievements in solving the problems of the chemical structure, biological function, and physiological activity of biopolymers and low–molecular–weight bioregulators, as well as in the elucidation of the molecular mechanisms of different life processes. The most striking achievements in bioorganic chemistry are discussed in this paper. However, this review discusses not only the general achievements in this field of science, but also research data obtained by scientists from the Pacific Institute of Bioorganic Chemistry, Far East Branch, Russian Academy of Sciences (Vladivostok, Russia), and the Institute of Physiology, Komi Science Centre, The Urals Branch, Russian Academy of Sciences (Syktyvkar, Russia). Particular attention is focused on comprehensive research into polysaccharides and biopolymers (bioglycans) and some natural glycosides that the author of this review has studied for a long time. The author has worked in these institutes for a long time and was honored by being chosen to head one of the scientific schools in the field of bioorganic chemistry and molecular immunology.

## INTRODUCTION

The chemical structure of biopolymers and bioregulators with low molecular weight, their biological activity, and their role in the organism are what bioorganic chemistry studies. Bioorganic chemistry also studies the physiological effects of different chemicals isolated from a particular source in a human or nonhuman organism. According to N.E. Spichenkov and V.E. Vas’kovsky [1], the term “bioorganic chemistry” was first mentioned in 1967 in an article by M.M. Shemyakin and A.S. Khokhlov [2]. All aspects of bioorganic chemistry were thoroughly reviewed by Yu.A. Ovchinnikov in his classical work [3] widely used by specialists in this scientific field.

Since the middle of the 20th century, bioorganic chemists have been interested in marine organisms [4–6], including seaweeds, marine animals, and bacteria. Many marine organisms are sources of unusual secondary metabolites with peculiar structures and unique properties [7]. Studies of the polysaccharides of seaweeds began in the 19th century and successfully continue today [8].

**Fig. 1:**
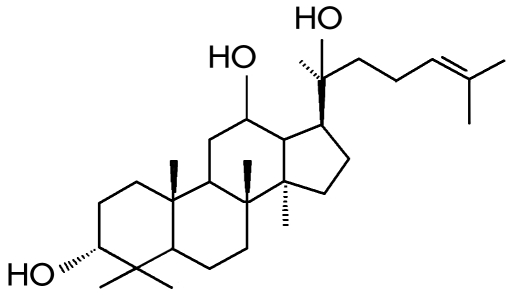
Fig. 1. Betulafolientriol from the leaves of birch and alder, one of the panaxosides’ genines, and the triterpenic glycosides of ginseng.

Bioorganic chemistry plays a crucial role in the development of pharmacological drugs, nutritional supplements, pharmacological chemistry, and medicine. This scientific branch rests on the basis of the chemistry of the natural compounds used in ethnomedicine, which has existed for thousands of years and helps in the choice of biological compounds as origins and sources of valuable biopharmaceuticals, nutritional supplements, and drugs. A detailed review of low–molecular–weight bioregulators of different types was recently published by scientists from Irkutsk [9–11]. Thus, in a historical perspective, a structural analysis of the ginseng and trepang extracts [4] used in Chinese medicine as active biostimulators shows that both extracts contain similar low–molecular–weight ingredients (bioregulators), which are triterpene glycosides lacking carbohydrate branches (aglicons and genins). Similar compounds were also found in birch and alder leaves, which in some cases can serve as raw material for the artificial synthesis of an effective agent of ginseng (*Panax gingeng*, C.A. May) and its glycosides (panaxosides) (Fig. 1). To trigger the physiological activity of these glycosides, a carbohydrate component (glycon) is needed, and it must contain oligosaccharides and comparatively short hydrocarbon chains.

## Plants and fungi polysaccharides

Plants are known to contain polysaccharides with long carbohydrate chains, linear or branched. Such polysaccharides make up 80% of the chemical compounds of the cell. They have different functions in the cell; many of them possess a marked physiological activity. Physiologically active polysaccharides contain glycuronic acids in their structure: mainly D–galacturonic and D–glucuronic acids. Plant polysaccharides such as pectic substances, gums, and mucilages [12, 13] demand special attention. Alginic acid, the main polysaccharide of marine brown seaweeds, consists of D–mannuronic and L–guluronic acids [14].

**Fig. 2:**
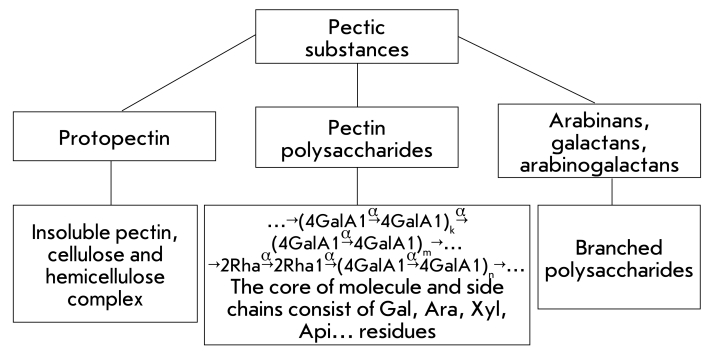
Components of pectic substances.

For a long period of time, pectic substances, which are the principium of so–called plant dietary fibers and biopolymers with high and polypotent biological activity [15], have been the object of special attention from researchers. The 1,4–linked residues of α –D–galacturonic acid form the backbone of pectic substances. The linear regions of this chain are interlinked by L–rhamnose residues involved into the chain by 1,2– α –glycoside linkages. The side chains of different lengths are made mainly of the arabinose and galactose residues which attached to the rhamnose residues. Pectic substances are considered a complicated polysaccharide complex composing the basis of the plant cell and including protopectin, an insoluble pectin complex with cellulose and hemicellulose; pectin polysaccharides of irregular structures; and concomitant branched polysaccharides: arabinans, galactans, and arabinogalactants (Fig. 2) [16].

**Fig. 3:**
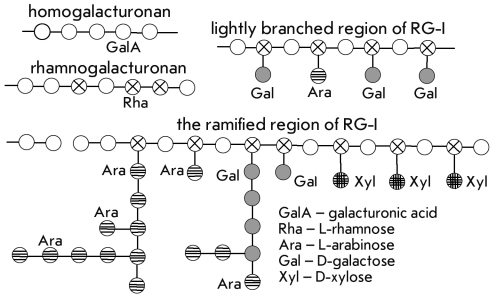
General scheme of the structure of pectin polysaccharides.

A general scheme of pectin polysaccharides is shown in Fig. 3 [16, 17].

Pectin polysaccharides were shown to possess a wide spectrum of physiological activity [17–19]. Therein, we should first mention their immunomodulating effect (phagocytosis stimulation); pronounced antiulcerous and antidotal action; and the ability to remove salts of heavy metals, organic toxins, and poisons from the organism. This is why, in hot shops and during chemical production, workers usually receive some pectins instead of milk as a preventive antidote. Apple pectins are also good for human health. Regular apple consumption (one in the morning and one in the evening) can prolong a human’s life by 10 years. Apple jam is supposed to be healthier because of its higher relative pectin content when compared to fresh apples. Galacturonans lacking side chains were found to possess pronounced antiinflammatory activity [20].

Several fungi should be mentioned, which are the sources and origins of other physiologically active polysaccharides. Lentinan and pachimaran are of great interest to researchers. Lentinan was first isolated by a Japanese research team headed by G. Chihara in 1969 [21] from the mushroom * Lentinus edodes * , which is widespread in the Pacific region and popular in Japan. Another active fungi glucan, called pachiman, was isolated from fungus *Poria cocos * [22, 23]. The mild periodate oxidation of pachiman leads to the generation of the more active pachimaran. Moreover, the latter fungus is a source of a series of physiologically active 1,3– β –D–glucans [24, 25]. Lentinan and pachimaran belong to the group 1,3– β –, 1,6– β –D–glucans, the backbone of which consists of D–glucopyranose residues linked by β –1,3–glycosidic linkages; the side chains of β –D–glucopyranose are attached to the backbone by the β –1,6–glycoside linkage [21–26].

Both polysaccharides possess high antitumor activity, which, as was shown by Chihara and other Japanese researchers, can be explained directly by their immunomodulating effect on the immune system of an organism with a tumor [21, 27, 28].

Thus, lentinan almost completely breaks the growth of a number of experimental tumors such as Gauss sarcoma, Ehrlich carcinoma, etc. Lentinan’s antitumor activity was shown to be caused by stimulation of T–lymphocyte killers, while no effect on B–lymphocytes and, thus, on the antibody development (humoral response) was observed. The high molecular weight of the fungi glucans (1 MDa) was shown to be a significant factor of their immunomodulating activity. However, the partial removal of the side chains of lentinan without a significant decrease in its molecular weight failed to affect immunomodulating activity; moreover, the transformation of pachiman into pachimaran leads to a noticeable increase in physiological activity [27].

Lentinan is widely used in medicine as an effective means for preventing and curing a number of malignancies.

The fungi of different species of * Ganoderma * genus and first of all of * G. lucidium * , which is widely used in ethnomedicine in Japan and China in particular, are widespread sources of 1,3– β –D–glucans [29, 30]. Over the last several years, comprehensive research into this group of fungi has been carried out [30–35]. 1,3– β –D–glucan was found to be one of the main components of * G. lucidium* [35], which possesses immunostimulating activity and, first and foremost, intensified phagocytosis and increased interleukin–1 (IL–1) production. Moreover, 1,3– β –D–glucans were shown to increase the production of an intensive immunomodulator, interferon (IFN– γ ) [36]. Therefore, fungus extracts are used to cure the impaired immune system, digestive tract ulcer, and cancer. A preparation based on the dry powder of *G. lucidium * , particularly, is used to cure sarcoma [37].

**Fig. 4:**
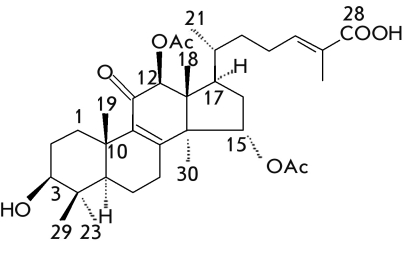
Ganodermic acid: the lanostanic triterpen of G. lucidum, G. applanatum.

Moreover, lanostanic triterpenoids found and isolated from different species of fungi [38, 39], ganodermic acid in particular, are being studied (Fig. 4). The presence of ganodermic acid in the aqueous ethanolic extract of * G. lucidium * was found to cause various physiological activities. The Ling–Zhi preparation based on * G. lucidium * (aqueous ethanolic extract) produced in China is used to cure nerve diseases, insomnia, vertigo, asthma, and other allergic manifestations [39].

A large body of data concerning the structure and physiological activity of 1,3– β –D–glucans from a number of fungi has been accumulated over the last several years [40–43].

## Bioglycans of marine invertebrates

The substantial bioglycan group of immunomodulators representing carbohydrate–protein biopolymers with a branched D–glucan was isolated from marine invertebrates. Branched D–glucan forms stable noncovalent bonds with the protein component representing lectin, which specifically binds polysaccharides. Immunomodulating activity was shown to need the presence of both bioglycan constituents [44, 45].

We have systematically studied many different marine invertebrates [45], and almost all of them were found to contain immunomodulating bioglycans. Our results led us to conclude that marine invertebrates produce immunomodulating bioglycans, which are responsible for their well–known resistance to arising neoplasma [45].

Mytilan is the most thoroughly studied immunomodulating bioglycan; it is isolated from different species of mussels of the Mytilidae family ( * Crenomytilus grayanus, Mytilus edulis, * and * M. galloprovinciales * ), ** which are widespread in the seas [44, 45]. Mytilan significantly increases phagocytic activity and factors of the humoral immunity. This substance is obtained from the so–called mussel juice, which was considered as waste in processing mussel as food stuff [6, 44, 45].

The property of mytilan behind the increase in the immune response to influenza has attracted the most interest. Because of that, mytilan has been widely used for influenza prevention and curation, especially at the early stage of the disease. The antiviral effect of mytilan is due to its ability to increase the biosynthesis of endogenous interferon, which plays a determinative role in the organism’s resistance to viral infection. Interestingly, mytilan has been found to double the mean lifetime of experimental animals [6, 45].

## Lypopolysaccharides of gram-negative bacteria

Since the middle of the 1930s, the attention of researchers has focused studies of the structural features and physiological activity of the antigens of gram–negative bacteria, the so–called O–somatic antigens, representing lipopolysaccharides (LPS) [46].

In the beginning, the French scientists A. Boivin and L. Mesrobeanu [47] isolated the immunogenic lipopolysaccharide–protein complex (LPPC) which is known up to now as the Boivin antigen [48]. Later, a huge number of works were devoted to the study of the structure and properties of LPS, which were not only O–somatic antigens of gram–negative bacteria, but also active endotoxins [49;]. LPS were found to consist of three main regions bound to each other as follows:

Lipid A – Core of the macromolecule – O–Specific polysaccharide.

A significant contribution to the study of the structure and properties of LPS has been made by the German Westphal–Luderitz school in Freiburg (FRG) since the 20th century. Since the 1960s, studies of the structure and properties of LPS have multiplied. In 1970 [50] at the Pacific Institute for Bioorganic Chemistry (Vladivostok), we started studying the antigenic composition of the pseudotuberculous microbe * Yersinia pseudotuberculosis * which causes a specitic disease, the so–called Far East scarlatinous fever (in Primorsky region). Research of LPS continues up to now. The most well–known works devoted to the study of LPS were carried out by the N.K. Kochetkov’s scientific school [51] especially by his pupil Yu.A. Knirel [52].

The structural features of the numerous LPS of gram–negative bacteria have been elucidated as a resut of these Research. Lipid A was shown to be responsible for the endotoxic properties of LPS, namely to cause the main symptoms of various diseases. The pattern of the immune response is determined by the O–specific polysaccharide, which is a matrix for the antibody production and differentiation. Therefore, this polysaccharide is in charge of the organism’s immune response during the development of the disease. The structure of O–specific polysaccharides can vary significantly. They have been found to contain the residues of many unusual monosaccharides. Such monosaccharides are often located at the terminal points of the macromolecule, and they determine its serospecificity, being immunodeterminant or immunodominant sugars [51–53].

As a rule, LPSs are not used for curation because of their high toxicity and, thus, low therapeutic index. However, on the basis of LPSs, many diagnostic techniques have been developed which make it possible to diagnose a disease at the earliest stage. Moreover, O–specific polysaccharides often possess an immunoadjuvant property; i.e., they can boost the effect of a vaccine against a certain disease [52, 54].

The LPSs of blue–green algae known also as cyanobacteria appear to hold much promise. Such LPSs are nontoxic, while they possess pronounced immunoadjuvant properties; on their basis we developed a powerful adjuvant lacking the side effects characteristic of classic adjuvants, such as the Freund’s adjuvant, which often causes abscess to develop at the injection site [55].

## Oncofetal antigens

The oncofetal antigens discovered by Yu.S. Tatarinov and G.I. Abelev in the beginning of the 1960s [56, 57] are of great research interest today [58–61]. Oncofetal antigens appear in the human organism during prenatal development, making the organism tolerant to these antigens. Later, they disappear and can appear again only during the development of an oncological disease. Thus, the cancer cell hides from the immune system of the host organism, which in this case cannot recognize the cancer cell as a foreign antigen. Therefore, oncofetal antigens are important markers of cancer (neoplasma) and are used to reveal tumors at the early stages of their development [44, 62, 63].

In this relation, the carcino–embryonic antigen (CEA) [63, 64], which does not differ in high specificity and is not revealed in the bodily fluids of a healthy human, is of great interest. Its appearance and accumulation in blood (more than 5 ng/ml) evidence the presence of almost any oncological disease in the organism, like neoplasias of the digestive tract and respiratory system or carcinoma of the breast, head, or neck [63]. Like all the oncofetal antigens CEA is a complicated glycoprotein, the protein part of which plays the determinative role. A study of its spatial structure showed that the carbohydrate moiety stabilizes the CEA conformation [64, 65].

It should be noted that many oncofetal antigens possess high enough, although not absolute specificity. Among them, the prostate–specific antigen (PSA) is one of the main markers of prostate cancer (PC), namely adenocarcinoma of prostate [66]. The PC is a male tumor, hormone–dependent at its early stages (androgen–dependent) [67–70], and widespread among men (second after lung cancer). Later, PC transforms into an androgen–independent metastasizing stage. Its lethality is usually due to metastases in bones and lymphnodes, as well as to switch of the tumor into the androgen–independent stage of tumor growth, which aggravates the process curing PC. Any effective curation of PC with metastases is absent in the present time. This disease is especially widespread among men in the United States. Official data show that PC was responsible for the death of more than 37,000 men in 1999 and about 30,000 men in 2005 in the U.S.A. Studies of PC are being pursued intensively there. An analysis of the PSA level in blood is widely used for PC diagnostics. Though this test often yields a false–positive reaction, it accurately determines the risk level. PSA is a single–stranded glycoprotein containing 240 a.a. with a molecular weight of 33–34 kDa [66]. There are several markers for the existence of PC, nevertheless an exact diagnosis remains difficult, although modern techniques for measuring PSA in blood have recently been developed [71–73].

**Fig. 5:**
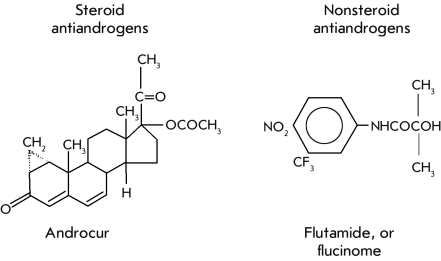
The inhibitors of transformation of testosterone into dihydro testosterone.

A cure for PC includes a sharp increase in physical activity, leading to the normalization of metabolic processes and to decongestion [69, 71]. Pharmaceutical treatment is based on the use of antioxidants, anti–inflammatory compounds, and antiandrogens (Fig. 5) inhibiting testosterone transformation into dihydrotestosteron. Antiandrogens are important when curing at the first androgen–dependent stage of the disease [66, 74–79].

**Fig. 6:**
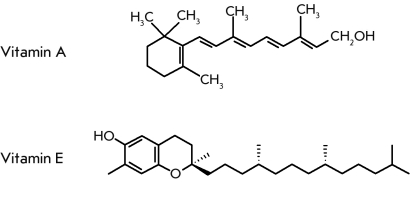
Vitamins A (retinol) and E (α-tocopherol), powerful antioxidants used for curing oncological diseases.

**Fig. 7:**
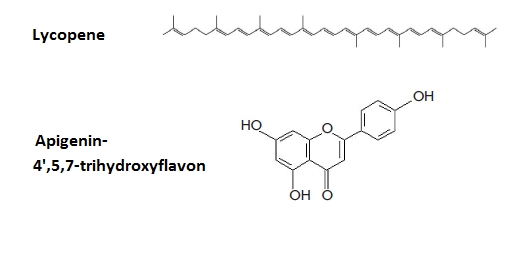
Antioxidants used for curing oncological diseases.

Strategies for curing any tumor, PC in particular, include the wide use of antioxidants. Increased oxygen consumption is a characteristic of tumor cells. Therefore, antioxidants, for instance, apigenin, an active component of green tea, effectively block tumor development (Figs. 6, 7).

Licopene (Fig. 7), a lipophilic hydrocarbon with a linear chain consisting of 40 carbon atoms and containing 11 conjugated and 2 nonconjugated olefinic linkages, possesses high antitumor activity [80]. Fresh tomatoes contain the licopene * trans * –form, which, after the tomatoes are processed, transforms into the * cis * –form. Licopene bioavailability for humans is 2.5–fold higher in tomato paste when compared to fresh tomatoes. Tomato consumption decreases the PC risk by 20%, while the use of tomato paste or tomato sauce leads to a 66% decline of this parameter. Therefore, the consumption of food containing tomato significantly decreases the risk of PC. Simultaneously, the cancer risk in the digestive tract, lung, and breast also declines [80].

Licopene has been shown to be an antioxidant and possesses a protective effect against lipid peroxygenation and DNA oxidative cleavage. These properties are behind the protective role played by licopene against tumors [81]. Licopene is used in medicine as a component of biologically active supplements such as licoprophit and licolam.

Thus, a number of medications exist that are used for curing oncological diseases. Many of them were offered by experts in bioorganic chemistry. However, more than any medications, the desire of a patient to resist the disease, constant cheertul mood, and an active way of life (which promotes metabolism normalization and intensifies immunity) are the main factors enhancing the organism’s resistance to oncological diseases.

## Active components of garlic

**Fig. 8:**
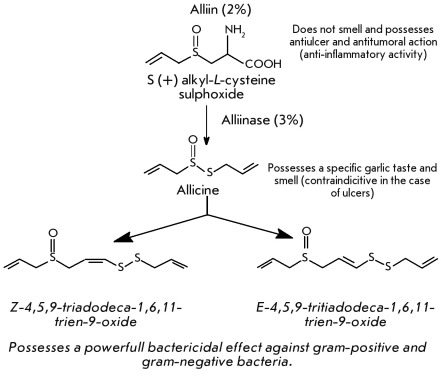
Active components of garlic (Allium sativum L.).

Garlic, * Allium sativum L * . (Fig. 8), contains very interesting compounds with different physiological functions [82]. The average content of alliin, a sulfoxide S(+)–alkyl–L–cystein, is about 2%. Alliin possesses a marked physiological action against gastric ulcer and is useful for curing oncological diseases due to its anti–inflammatory activity.

However, garlic contains about 3% of a highly active enzyme, alliinase, which strongly intensifies in the presence of atmospheric oxygen, as was demonstrated by Japanese researchers [83]. This explains the fast alliin digestion on grinding garlic leading to allicin and the formation of other sulphur compounds, which are responsible for the specific smell and taste of garlic. These compounds possess a powerful bactericidal effect against gram–positive (e.g., *Staphylococcus aureus * ) and gram–negative (e.g., * Salmonella sp.* ) microorganisms, but they markedly irritate mucous membranes and are contraindicated in case of gastric ulcer [83]. Sulphur compounds were also shown to decrease carcinogen–induced cancer development in several organs due to their high antioxidative activity and ability to stimulate the immune response [84].

## Flavonoids and alkaloids

Flavonoids are a large class of active natural compounds widespread in the plant world. They appear to be different derivatives of chromane and isochromane and are found in nature as glycosides and aglycons [9–11].

**Fig. 9:**
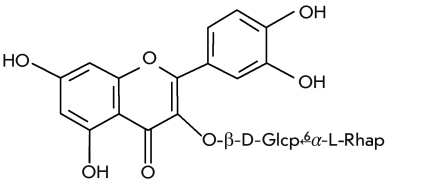
Rutin (vitamin P), quercetin’s rutinoside.

Quercetin bioside (Fig. 9), called rutin after the plant rue from where it was first isolated, can be such an example [9, 10]. Rutinose, a disaccharide, is a constituent of rutin. Rutin was isolated from buckwheat leaves and belongs to the group P of vitamins. In the mammalian organism, rutin strengthens blood capillaries and increases blood coagulation. The presence of the vitamin C increases the effect of rutin. Rutin is used for curing diseases connected with blood strokes caused by increased capillary fragility and a defect in the blood coagulation system. Such a physiological action is characteristic of many flavonoids, which more or less possess P–vitamin activity. 

 Flavonoids are powerful antioxidants and can bind free radicals damaging the cell walls of normal cells.

**Fig. 10:**
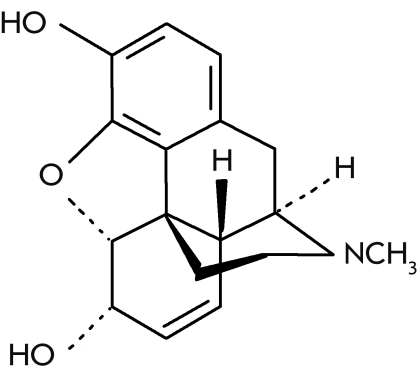
Morphine, poppy (*Papaver somniferum*) alkaloid, strong narcotic, and powerful analgesic.

Of all the other low–molecular–weight bioregulators, only alkaloids will be mentioned [3], which are various, highly active compounds containing nitrogen. Many of them are strong narcotics and analgesics. Morphine (Fig. 10), the main alkaloid of the poppy ( *Papaver somniferum * ), is one of the most famous alkaloids [3, 85]. Bioorganic chemists have elucidated not only the structure of this very complicated natural compound, but also succeeded in synthesizing it. In addition, ajmaline and allopinine are alkaloids with a strong antiarrhythmic effect.

## Poisons and toxins

Poisons and toxins, small doses of which cause the death of an organism, possess the highest physiological activity. The terms * poison * and * toxin* are very similar, though poisons are any toxic substance irrespective its origin, e.g., cianide, arsenic, and cobra poison. Toxins are poisons of a biological nature only [3].

Poisons and toxins are substances of the highest physiological activity and selectivity. Botulinotoxin, the protein toxin of the anaerobic bacteria * Clostridium botulinum, * is the most toxic among poisons and toxins [3, 86]. Other clostridium toxins possess the same high toxicity. A lethal dosage of botulinotoxin is equal to 10^–5^ μg/kg, while the toxicity of potassium cyanide is 10^4^ μg/kg. Botulinotoxin is synthesized by clostridia in strictly anaerobic conditions. This is a powerful neurotoxin blocking the transduction of neural impulses, which leads to paralysis and death. Humans and domestic animals are the most sensitive subjects to botulinotoxin. Humans are usually affected by botulinotoxin after eating poorly preserved food, like meat; fish; and, more frequently, agarics. Clostridium endospores are well preserved on unpeeled mushrooms. Endospores are stable to the tough condition of sterilization. During home conservation, conditions are often nonsterile, which promotes the survival and reproduction of clostridium endospores in an anaerobic medium, leading to the generation of the active form of clostridia, which produces botulinotoxin. Botulism is a severe disease and leads to death. This is why buying canned mushrooms of unknown origin and eating underboiled preserved mushrooms are not recommended. It is recommended to boil even homemade canned goods before eating to destroy the synthesized botulinotoxin in case it formed in the conserved product. Using anatoxin, i.e. a toxin treated previously with formalin, or using a serum against anatoxin gives good results in curing botulism at all stages of the disease’s development.

Cyclopeptide toxins of the death cup * Amanita phalloides * [3, 76], amanitine and phalloidin, are other powerful toxins of a protein nature which were first isolated as individual substances in 1937 by the German researchers F. Linen and G. Viland. Poisoning by death cup toxins is widespread. These toxins are distinguished by their prolonged latent period (up to 36 hours) as the liver is painlessly destroyed. By the time the symptoms emerge, any kind of cure appears to be ineffective, which explains the high percentage of mortality by intoxication. Interestingly, eating even one death cup can be fatal. At the same time, the death cup contains peptide antamadine, a 1 mg/kg concentration of which protects the organism from the destructive action of the toxin. Hepatoprotective medication (carsil, isolated from * Silybum marianum* and available in any pharmacy) has a positive effect, which is especially preventive against amanitine intoxication.

Among the nonprotein toxins, we will mention shortly toxins produced by marine organisms.

**Fig. 11:**
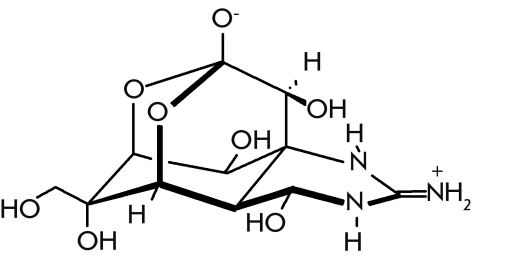
Tetrodotoxin.

Tetrodotoxin, contained in fish of the Tetraodontidae family (Fig. 11), is one of the most well–known nonprotein toxins. The most famous of the family goes by the names hogfish, swell fish, or puffer fish * Fugu niphobles * and * F. rubripes* . Tetrodotoxin was also isolated from the Corsican frog * Atelopus sp * ., the crab * Atergatis floridis * , and the Californian triton * Taricha torosa* .

The puffer fish is considered a delicacy in Japan. Tetrodotoxin is accumulated in different fugu organs, but mainly in roe, liver, and skin. Special chefs carefully remove these organs before cooking. However, in some seasons, tetrodotoxin accumulates in all of the fugu body, making it completely toxic, which often leads to fatal consequences. In some years, hundreds of people die in Japan after eating puffer fish. This is why the puffer fish is served only in special restaurants displaying signs that read “Want to try fugu? Write your last will.”

Tetrodotoxin is a powerful neurotoxin causing the paralysis of human and mammalian skeletal muscle, a sharp decline in blood pressure, and death by respiratory arrest. The lethal dosage is equal to 7 μg/kg.

Tetrodotoxin is widely used in laboratory practice to study the mechanisms of neural transduction.

**Fig. 12:**
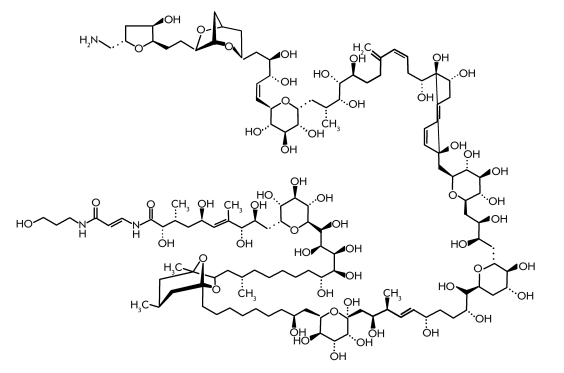
Palytoxin.

Palytoxin is another nonprotein toxin of marine origin with a complicated structure (Fig. 12). It was first isolated in 1971 by the American scientists R.E. Moore and P. Scheuer [87] from the soft corall * Polytoa toxica * . The results of numerous studies of palytoxin have been described in a detailed review [88]. While the toxin was being isolated by Drs. Moore and Scheuer, a conflagration appeared in the laboratory. Everybody who dealt with the toxin suffered; particularly the cardiovascular system of the researchers was injured. Consequently, this powerful toxin was named “messenger of Satan.” The complex structure of palytoxin was determined a decade later, in 1981, by two independent research groups, one from Japan headed by Prof. Y.Hirata [89, 90] and another by Prof. R.E. Moore [91]. The determination of the palytoxin structure became a significant event in bioorganic chemistry. Its molecule possesses a unique structure [3] (Fig. 12). A stereochemical study of palytoxins was successfully carried out by the above–mentioned groups of Japanese and American researchers [92, 93]. In spite of the existence of a huge number of possible stereoisomers, a group of Japanese researchers headed by Prof. Y. Kishi [94] in 1989 successfully synthesized several palytoxin derivatives. A comparison with the natural palytoxin showed that synthetic substances were identical to natural ones in biological activity, chromatographic behavior, and spectral characteristics obtained by NMR spectroscopy and mass spectrometry [94]. Several years later, in 1994, a palytoxin specimen completely identical to the natural one was synthesized [95]. However, the complete chemical synthesis of palytoxins includes 65 stages [96], which makes it impossible to use in every day practice. The physiological action of palytoxin is quite significant [3]; being injected intravenously, it is LD_50_ = 0.15 μg/kg for mice and 0.08 μg/kg for monkeys. The lethal outcome takes 5–30 minutes as a result of deep injury to the cardiovascular system and respiratory arrest. The toxic dose for different animals varies from 0.01 to 0.1 μg/kg of LD_50_ [96]. The toxic dose for human was not measured experimentally of course, though an extrapolation of the data obtained in studies on different animals makes it possible to conclude that it takes LD_50_ of about 0.04 μg/kg [97]. In spite of the high toxicity, palytoxin was found in a number of marine invertebrates [88, 97]. Interestingly, palytoxin in the sublethal dose demonstrated a high antitumoral activity.

In tropical seas, “red tides” are often observed causing the massive death of various marine organisms as a result of the intensive multiplication of toxic microalgae (dinoflagellates). These microalgae release a number of strong toxins, including the most powerful of the known nonprotein toxins, maitotoxin [98]. This toxin was first found in 1976 [99] in the intestine of the surgeonfish *Ctenochaetus striatus * as the main component of siguatera, the famous food toxin of dinoflagellates * Gambierdicus toxicus * , which is included in the food chain of herbivorous fish. The toxicity of maitotoxin is the highest, LD_50_ is equal to near 0.05 μg/kg for mice. Maitotoxin was first isolated as an individual substance in 1988 by a group of Japanese researchers headed by Prof. T. Yasumoto from Tochoku University [100]. The structure of this complicated substance, including the elements of stereochemistry, was elucidated in 1992–1994 [101] by this research group using spectral analysis, NMR spectroscopy, and mass spectrometry techniques, in particular. They also determined the absolute configuration of maitotoxin in 1996 [102]. The data obtained were confirmed later, when maitotoxin was artificially synthesized by a group of Japanese scientists headed by Y. Kishi [103].

These data demonstrate clearly that bioorganic chemistry has assumed a prominent place among the sciences that study basic life processes and the substances playing an active role in those processes. Bioorganic chemists continue to study the chemical structure of new biopolymers and low–molecular–weight bioregulators and to shed light on their biological functions and physiological activity. Special attention is focused on unveiling the interrelations between the structural features and biological activities of different chemical substances. Bioorganic chemists, together with biochemists, biotechnologists, physiologists, and doctors, are obtaining new results that help extend productive human life.

## ABBREVIATIONS

LPS: lipopolysaccharide
PSA: prostate specific antigen
LPPC: lipopolysaccharide-protein complex
CEA: carcino-embryonic antigen
PC: prostate cancer
